# Nanostructures Formed by Custom-Made Peptides Based on Amyloid Peptide Sequences and Their Inhibition by 2-Hydroxynaphthoquinone

**DOI:** 10.3389/fchem.2020.00684

**Published:** 2020-08-06

**Authors:** Radhika Mannem, Mohammed Yousuf, Lakshmaiah Sreerama

**Affiliations:** ^1^Department of Chemistry and Earth Sciences, Qatar University, Doha, Qatar; ^2^Central Laboratory Unit (CLU), Qatar University, Doha, Qatar

**Keywords:** amyloid fibrils, Aβ sheet mimics, SEM, THT, quinones

## Abstract

Extensive research on amyloid fibril formations shows that certain core sequences within Aβ peptide play an important role in their formation. It is impossible to track these events *in vivo*. Many proteins and peptides with such core sequences form amyloid fibrils and such Aβ sheet mimics have become excellent tools to study amyloid fibril formation and develop therapeutic strategies. A group of peptides based on amyloid peptide sequences obtained from PDB searches, where glycine residues are substituted with alanine and isoleucine, are tested for aggregation by SEM and ThT binding assay. SEM of different peptide sequences showed morphologically different structures such as nanorods, crystalline needles and nanofibrils. The peptides were co-incubated with HNQ (a quinone) to study its effect on the process of aggregation and/or fibrillation. In conclusion, this group of peptides seem to be Aβ sheet mimics and can be very useful in understanding the different morphologies of amyloid fibrils arising from different peptide sequences and the effective strategies to inhibit or anneal them.

## Introduction

Amyloid formation has been proven to be the underlying trigger for a number of protein deposition diseases like Alzheimer's, Parkinson's, diabetes, β2m-amyloidosis, carpel tunnel syndrome, destructive arthropathy, transthyretin amyloidosis, transmissible spongiform encephalopathy, hemodialysis related amyloidosis etc. (Bucciantini et al., 2002; Bu et al., 2007; Siddiqi et al., 2018). The self-assembly of amyloid proteins into fibrils has been found to be the basis for the pathogenic process in all these diseases (Bucciantini et al., 2002; Cuvalevski et al., 2012). Functional proteins result from the folding of proteins into specific conformations. But some proteins are found to misfold into amyloid fibrils which seems to be the primary cause in many diseases mentioned above. Much remains to be elucidated regarding the molecular mechanisms involved in the amyloid aggregation, particularly the different entities formed down the cascade and the significance of peptide sequences in the formation of these entities (Tjernberg et al., 1999; Ivanova et al., 2006).

Amyloid β protein (Aβ), one of the key players in Alzheimer's disease, is generated from amyloid precursor protein by sequential proteolysis (Bensney-Cases et al., 2012). During this stepwise cleaving, many peptides with different C-termini are formed among which Aβ (1-40) is most common and, also Aβ (1-42) that forms fibrils and appears to be closely linked to neurodegenerative diseases (Dolphin et al., 2006; Cuvalevski et al., 2012). Protein specific amyloidogenic core sequences are responsible for cross β formations that make up amyloid fibrils (Lakshmanan et al., 2013). These core sequences called the self-recognition elements (SREs) within β amyloid seem to play an important role in its aggregation into oligomers and fibrils (Kumar et al., 2015). The Aβ fibrils have a higher content of β sheets formed due to molecular self-assembly of Aβ. Three factors that drive molecular self-assembly are hydrogen bonding, hydrophobicity and electrostatic interactions (Hong et al., 2003). However, protein folding strongly depends on hydrogen bonding and on the hydrophobic interactions which seem to be the possible mechanisms that can bring about the self-assembly of the peptides investigated in this study. These peptides are designed based on the fibril forming/amyloidogenic sequences of Aβ 42 with variations in sequences.

Many of the amyloid proteins have more than one fibril forming core sequences (Sawaya et al., 2007). These core sequences usually 4–7 or 6–8 amino acids long are found to be pivotal in forming the nucleation sites for amyloid aggregation (Mehta et al., 2008) and also capable of assembling into fibrils by themselves that are similar to those formed by the whole proteins in all aspects. Therefore, these core sequences can serve as relatively simple model systems to study amyloidosis, particularly the early intermediate structures which are increasingly postulated as the toxic species resulting from amyloid aggregation. Further, they can be used to develop therapeutic drugs that can target the amyloid formation and arrest it at the earlier stages.

A study on the fragment 20–29 of amylin indicated that the properties of amino acids that promote the aggregation of peptides are hydrophobicity (so that the peptide chain folds on itself, forming a β sheet), planar geometry and β sheet propensity. Studies have demonstrated that valine, isoleucine and alanine have β sheet propensity due to their hydrophobicity and hydrogen bonding characteristics (Han et al., 2011; Cui et al., 2014). Whereas formation of direct π-π interactions involving phenylalanine do not seem to be important in promoting amyloid aggregation (Stankovic et al., 2017).

Proteins and peptides not associated with amyloid diseases are found to have the propensity to aggregate into fibrils *in vitro*, very similar to those found in amyloid diseases (Bucciantini et al., 2002). Peptides are capable of assembling into amyloid fibrils similar to those formed by the whole proteins. The amyloidogenic peptides can form dimers and trimers that can undergo nucleation into oligomers and then elongation into needle like microcrystals or fibrils that can be tubular or ribbon like or flexible helices (Ivanova et al., 2006; Bensney-Cases et al., 2012). The amyloid proteins are also found to have different fibril forming segments that form different structural phenotypes as shown in GGVVIA and MVGGVV sequences of amyloid β that form parallel and antiparallel β sheets, respectively (Colletier et al., 2011). The fibrils, which are thus polymorphic, might also have structures from different segments in a single entity. Different peptide sequences are widely studied to understand the mechanisms and factors that govern the amyloid fibril formation which is found to be common in various amyloid diseases. Another hallmark of amyloid diseases is that the native proteins transform into ordered oligomers and fibers from β sheet rich structures. Developing model systems of β sheets and the resulting amyloid entities is a plausible approach to study the bewildering variety of amyloid structures (Cheng et al., 2012). The hydrogen bonding between the peptide backbones initiate the monomers to pack longitudinally into β sheets (Zhao et al., 2015). Attractive forces between the amino acid side chains promote the lateral stacking of these β sheets which is constrained at the same time by the repulsive forces and the natural twisting of the β sheet themselves. These interactions, geometrical constraints and their interplay result in different forms of peptide self-assemblies.

Thus, there is a need to evolve therapeutic strategies aimed at different amyloid aggregates that are possible from amyloidogenic peptides with different sequences as considered in this study.

The protein specific amyloidogenic core sequences are demonstrated to be responsible for cross-β formations that make up amyloid fibrils and these core sequences are found to be 4–7 amino acids long (Lakshmanan et al., 2013). It was found that a peptide with 4–7 amino acids is sufficient to form a fibril that can trigger the folding of the entire protein (Sawaya et al., 2007). A group of aliphatic peptides designed based on core sequences of amyloidogenic Aβ42, human amylin and human calcitonin showed that aromatic residues are not necessary for the formation of cross β aggregates as mentioned earlier (Lakshmanan et al., 2013). One such example is GA_6_, which is an aliphatic sequence from the transmembrane domain of the Aβ and found to have high propensity for amyloid aggregation. Scientists generated synthetic peptides also based on the natural amyloid forming proteins that are found to assemble lengthwise to produce amyloid fibrils of few nanometers width and several micrometers length (Chamberlain et al., 2000; Smith, 2012). Computational studies have suggested that the presence of glycine and aromatic amino acid residues in the protein sequences readily promote aggregation and/or fibrillation (Gill, 2014; Stankovic et al., 2017). However, it was found from PDB searches (Ninkovic et al., 2017; Stankovic et al., 2017) that in the sequences containing only aliphatic residues, e.g., AIIGLM, MVGGVVIA and NKGAII, the interactions between the β sheets of tetramer models were similar in strength to the energies associated with the aromatic residues in the aromatic sequences of KLVFFA and NFGAILS. Both aromatic- aromatic and aliphatic-aliphatic interactions between the tetramers of these aromatic and aliphatic sequences were slated to play an equally important role in amyloid aggregation (Stankovic et al., 2017). Our group investigated a cluster of PDB based peptides where glycine residues in the sequences based on Aβ 42 are substituted with more hydrophobic alanine and isoleucine, for amyloid formations (Table 1). These peptides can be prospective models to study the amyloid cascade mechanism at molecular level and for testing therapeutic compounds that can inhibit amyloidosis.

**Table 1:** List of peptides identified based on PDB searches.

**Amyloid peptides identified by PDB/QM and MM studies**	Peptides in which Gly substituted with Ala^*^	Peptides in which Gly substituted with Ile^*^
MVGGVV	MVAAVV (P1)	MVIIVV (P2)
GGVVIA		IIVVII (P7)
AIIGLM	AIIALM (P4)	
MVGGVVIA	MVAAVVIA (P5)	MVIIVVIA (P6)
GAIIGL	AAIIAL (P3)	

* *All peptides listed above were commercially synthesized (JPT Peptide Technologies GmbH, Berlin, Germany)*.

Detailed information about various oligomeric forms of Aβ is also needed to comprehend their pathological roles or for testing compounds that inhibit their oligomerization or prevent their entry into the cells or their movement from cell to cell (Breydo et al., 2016). This study focuses on the structures formed by a variety of peptide sequences and evaluate the effect of quinone (HNQ) on these structures as examined by Scanning Electron Microscopy (SEM), thioflavin T (ThT) binding assay, and FT-IR spectroscopy.

## Materials and Methods

### Materials

2-Hydroxy-1,4-naphthoquinone (HNQ) and 1,1,1,3,3,3-Hexafluoro-2-Propanol (HFIP) were purchased from Sigma Aldrich Chemical Co, St Louis, MO, USA. SensoLyte ThT dye kit was purchased from AnaSpec. Inc, Fremont CA, USA. All other chemicals were available locally and were of analytical grade.

### Peptide Samples

The peptides with the sequences MVAAVV (Peptide 1 or P1), MVIIVV (P2), AAIIAL (P3), AIIALM (P4), MVAAVVIA (P5), MVIIVVIA (P6), and IIVVII (P7) were custom made and procured from JPT Peptide Technologies GmbH (Berlin, Germany). To evaluate the differences in amyloid structures formed, if any, according to the sequence variations, the middle AA amino acids in P1 are replaced with II in P2. Similarly, the middle sequence II is flanked by AA at the amino terminus and AL at the C terminus in P3. In peptides P5 and P6, IA is added to the amino acid sequences in P1(MVAAVV) and P2 (MVIIVV), respectively, at the C terminus (Table 1).

### Preparation of Peptide Samples for Storage

The aliquots of the peptides were prepared following the protocol of Stine et al. (2011). Briefly, 2 mg of peptides were dissolved in appropriate volume of 1,1,1,3,3,3-Hexafluoro-2-Propanol (HFIP, Sigma Aldrich Chemical Co, St Louis, MO, USA) to obtain 2 mM concentration. An aliquot of 200 μl of each peptide was dispensed into microcentrifuge tubes and left in the laminar flow hood for the evaporation of HFIP. Following this, the aliquots were taken for SpeedVac (Thermo Fisher Scientific, USA) to vaporize the HFIP completely leaving a thin layer of peptide on the micro centrifuge vial. They were then stored in glass jars with desiccant at −20°C till they are used for further analysis.

### Scanning Electron Microscopy (SEM)

The peptide aliquots (0.2 mM) were prepared in 0.4 mM sodium bicarbonate buffer, pH 11 and incubated at 37°C with continuous shaking for 5 days. The sample solution (10 μl) was loaded onto the SEM stubs and dried by stepwise ethanol treatment to preserve the peptide nanostructures (Mammadov et al., 2012). They were then sputter-coated with gold and were imaged using a SEM (JEOL, Boston, MA, USA).

### ThT Binding Assay

Fluorescence changes due to specific binding of Thioflavin T dye to peptide aggregates/fibrils were measured as per the protocol provided by the manufacturer (SensoLyte ThT dye kit, AnaSpec. Inc, Fremont CA, USA). Aβ (1-42) provided with the kit was used as control and prepared as per the instructions in the protocol provided. The peptide solutions (2 mM concentration) were prepared with the assay buffer (supplied with the kit). Ninety microliter of each of the peptide solution was added in duplicate to the wells of microplate. Freshly prepared ThT solution was added to the peptide solutions to a final concentration of 0.2 mM. The florescence (excitation at 440 nm and emission at 484 nm) due to ThT binding was recorded using a Spectramax microplate reader (Molecular Devices, San Jose, CA, USA). The plate was incubated at 37°C in the microplate reader and shaken for 30 s before each reading and the readings were taken at an interval of 15 min.

### FT-IR Spectroscopy

The peptide samples (0.2 mM) were prepared in 0.4 mM sodium bicarbonate buffer, pH 11 and incubated at 37°C with continuous shaking for 5 days. The FTIR spectra of the peptide samples were recorded in the range of 4,000–400 cm^−1^, using Spectrum 400 FTIR spectrometer [PerkinElmer equipped with UATR (Universal Attenuated Total Reflectance)] and DTGS detector.

### Treatment of Peptides With Inhibitor (HNQ)

HNQ (50 μM) was mixed with peptide samples (100 μM) and incubated at 37°C for 3 days with continuous shaking for aggregation inhibition experiments (Bermejo-Bescos et al., 2010). The aggregation of peptides was then imaged by SEM as described above.

## Results

### Nanostructure of Peptides

**Figure 1:**
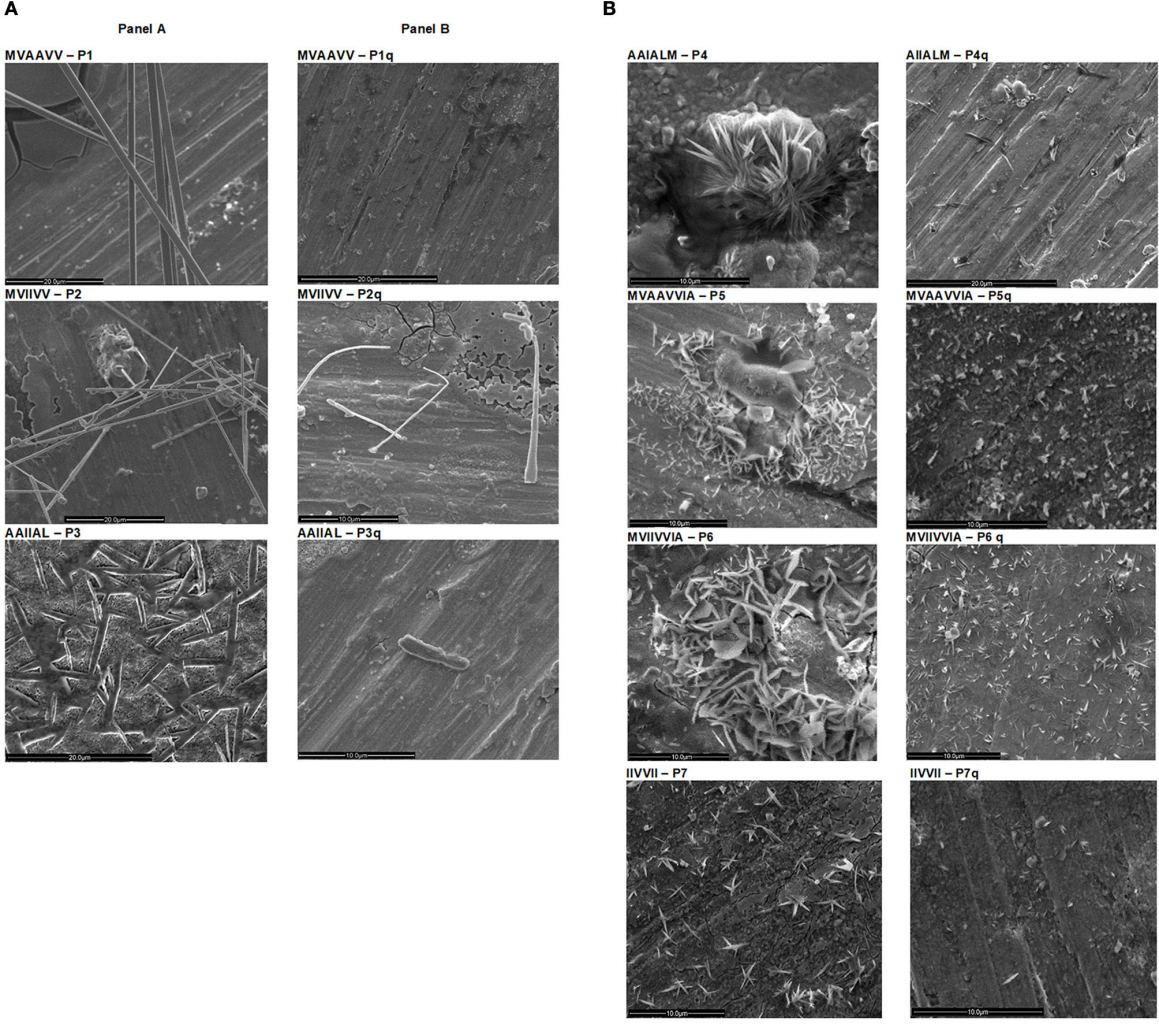
Scanning Electron Micrographs of peptides (P1–P7) before and after treatment with HNQ. SEM of peptides P1–P7 incubated at 37°C for 5 days is shown in **(A)**. P1, P2, & P3 show nanotubes (20 μm scale). P4 shows a thick mat of crystals and P5, P6, & P7 show crystals (10 μm scale). The SEM images of peptides treated with HNQ are shown in **(B)**. After HNQ treatment peptides P1, P2, P4, & P6 show much shortened aggregates (P1q, P2q, P4q, & P6q); P3 is almost dissolved (P3q); P5 and P7 showed amorphous aggregates (P5q & P7q) (scales 10–20 μm).

Studying, in particular, the elongation of preformed fibrils in a multistep aggregation process of α-synuclein, it was established that even within the fibril population of a single amyloid protein the size of the fibrils can be highly heterogeneous (Pinotsi et al., 2014). The cluster of peptides tested in this study formed different types of aggregates and they grew to different lengths during the same period of incubation, i.e., the aggregates formed by different peptides were hugely heterogenous. For example, P1 (MVAAVV), P2 (MVIIVV), and P3 (AAIIAL) of our peptide cluster show long nanotubes (Figure 1A) similar to those formed by Aβ (16-22) peptide sequence at pH 2, which were proved to be helices of cross β sheets (Perutz et al., 2002; Dong et al., 2006). The formation of nanotubes was attributed to a different sequence alignment in tubes from that of fibers formed by the same peptide. On the other hand, peptides P4 (AIIALM), P5 (MVAAVIA), P6 (MVIIVIA), and P7 (IIVVII) formed microcrystals (Figure 1A). Earlier, microcrystals were shown to be formed by VQIVYK from tau, NNQQ from Sup35, GGVVIA from amyloid-β, and LYQLEN from insulin (Sawaya et al., 2007). These peptide segments were found to form both microcrystals and fibrils that were anticipated to have similar atomic arrangements. The principal diffracting features of protein fibrils and the corresponding microcrystals formed by the same peptide were also closely similar. The segment G_6_V derived from αβ crystallin was also found to form microcrystals (Laganowsky et al., 2012). The fiber-forming Aβ segments also showed microcrystal and fibrillar structures (Colletier et al., 2011). So also the peptides in this study which are designed based on some of these Aβ segments (Aβ_35−40_, Aβ_29−34_, Aβ_30−35_, and Aβ _35−42_) whose Gly residues are replaced with Val, Ala, and Ile.

### Thioflavin T Binding by the Peptides

**Figure 2:**
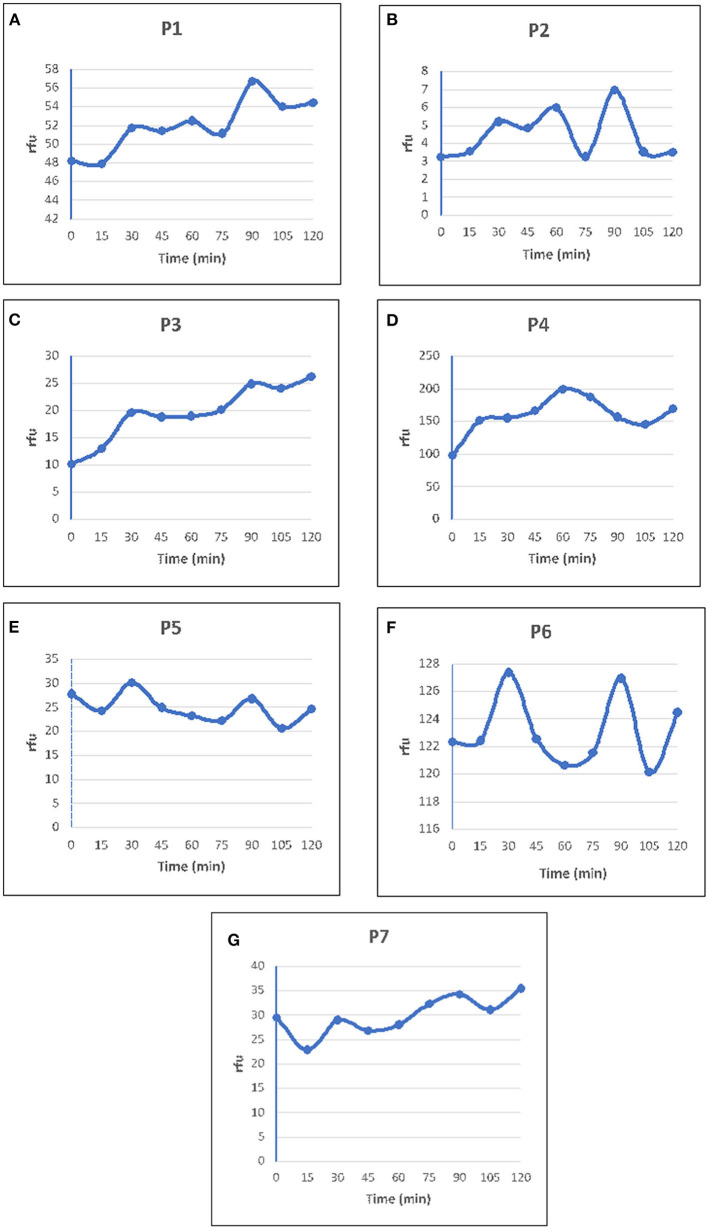
ThT binding assay of peptides P1–P7. To each of the peptide (2 mM) solutions freshly prepared Thioflavin T (ThT) was added to a final conc. of 0.2 mM. The fluorescence reading was measured at every 15 min intervals until no further increase in fluorescence was observed. Relative Florescence Units (RFU) plotted against time for each of the peptides P1–P7 as depicted in **(A–G)**. Peptides P4 and P6 showed higher intensities compared to other peptides. RFU is the difference between the fluorescence of ThT without the peptide, and the fluorescence of the ThT with the peptide, in the reaction mixture.

ThT is used to detect amyloid fibrils in biological samples and analogs of ThT are even used as probes for amyloid imaging by Positron Emission Tomography and Single Photon Emission Computed Tomography (Maezawa et al., 2008). The cross β structure of most amyloid fibrils can accommodate ThT which is routinely used to recognize them *in vitro, ex situ* and in histological samples. All the peptides tested in the present study showed an increase in ThT fluorescence (Figure 2), and the fluorescence intensities were more for peptides P4 and P6 (Figure 2) compared to other peptides. P4 and P6 showed thick network of crystals in SEM (Figure 1A). Though SEM images of P1, P2, and P3 show nanotubes (Figure 1A) and fibrils they show weaker intensities.

### FT-IR Spectra of the Peptides

**Figure 3:**
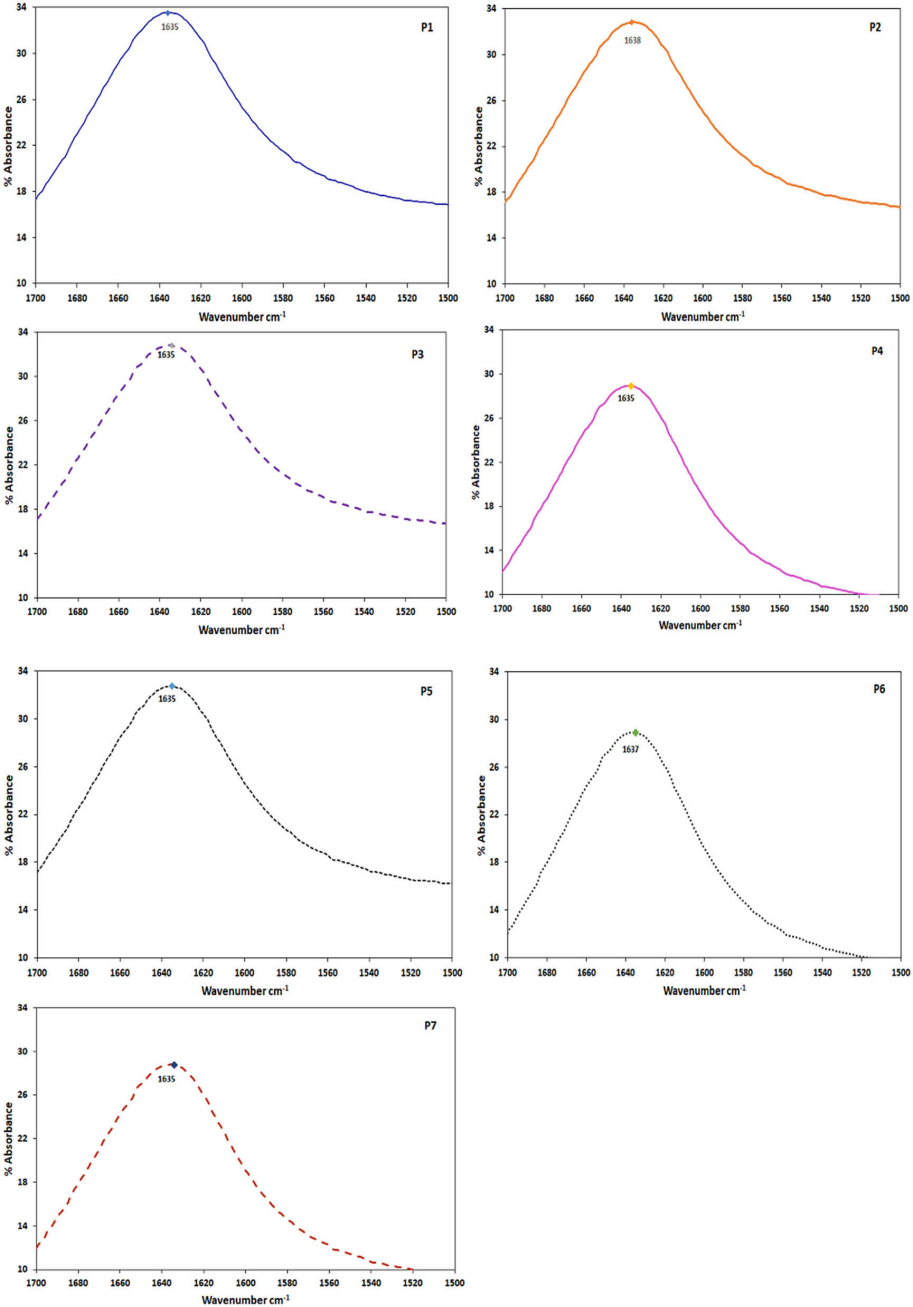
FTIR spectra of the peptide cluster (P1–P7). The peptides at 0.2 mM conc. were incubated for 5 days before taking the spectra. All the peptides showed a wavenumber max at ~1,635 cm^−1^.

Bands in the amide I region of IR spectrum from 1,643 to 1,615 and 1,692 to 1,697 cm^−1^ are due to β sheet conformations (Shivu et al., 2013). FT-IR analysis of our cluster of peptides showed bands between 1,634 and 1,638 cm^−1^ (Figure 3) which correspond to the amide I region, confirming the presence of β sheets in their aggregates. Though X-ray crystallography or Nuclear Magnetic Resonance (NMR) give detailed information about molecular conformations, FTIR is currently widely used as it gives a quick overview of the same. FTIR spectra of different amyloid fibrils from different sources showed bands in the amide I region. Amyloid fibrils from amyloid β showed at 1,628 cm^−1^, insulin at 1,632 cm^−1^ and a synthetic peptide ED (EDVAVYYCHQYYS) at 1,634 cm^−1^ (Shivu et al., 2013). A 9-residue peptide from the amyloid protein Sup35 formed amyloid like crystals and showed a band at 1,633 cm^−1^ typical of parallel β sheet (Balbirnie et al., 2001).

### Inhibitory Action of HNQ

The inhibitory investigations of amyloid aggregation usually employ transmission electron microscopy to visualize the effects of inhibitors at molecular level as applied in many studies (Bermejo-Bescos et al., 2010; Gong et al., 2014; Li et al., 2019; Scheidt et al., 2019). In this study, we co-incubated the amyloid peptides with HNQ (peptide: HNQ concentration = 8:1) for 3 days and took SEM images of treated samples. When treated with HNQ, peptides P1q, P2q, P4q, P5q, P6q, and P7q showed much reduced density of shortened aggregates that appeared disrupted (Figure 1B). They looked similar to the disaggregation of amyloid fibrils by capreomycin (Siddiqi et al., 2018). The mesh of fibers in peptide P3 incubated with HNQ disappeared (Figure 1B) as has been observed in similar studies on inhibitor action of 1,4-naphthoquinones (Gill, 2014), quinones (Gong et al., 2014), and other polyphenols (Ono et al., 2003), and copper (Mold et al., 2013) on amyloid formations. Amyloid β treated with white tea also showed amorphous aggregates whereas the overall density of aggregates was less in those samples treated with other types of tea (Li et al., 2019). Tubular aggregates of P3 appear to have dissolved as has been observed when amyloid aggregates were treated with curcumin (Yang et al., 2005).

## Discussion

### Polymorphism of Amyloid Nanostructures

The molecular dynamic simulations of Aβ (16-22) showed that the side chains on β-sheets of fibers had polar and nonpolar faces while the same side chains displayed on the β sheets of nanotubes had symmetric faces, which contributed to sheet lamination ensuing nanotubes (Mehta et al., 2008). In a comparison between the nanostructures formed by KI_4_K and KKI_4_, it was concluded that the electrostatic repulsive forces from 2 lysine residues at the N-terminus weaken the hydrophobic adhesion between the isoleucine residues. Thus, KKI_4_ forms thin nanofibers whereas in the absence of such strong repulsion KI_4_K forms wider helical ribbons that wrap up into nanotubes (Zhao et al., 2018). In the absence of polar residues in our peptide cluster, we hypothesize that a similar lack of electrostatic repulsions and the unaffected hydrophobic interactions of the amino acids on one peptide chain with those of the next chain, resulted in the nanotubes. Much earlier X-ray studies showed that amyloid fibers formed by Aβ of AD and synuclein protein involved in Parkinson's disease, are nanotubes with a cavity filled with water (Perutz et al., 2002). A segment of α β crystallin which is also amyloidogenic was reported to form cylindrical barrel like structures comparable to the structures formed by Aβ of AD (Laganowsky et al., 2012).

The differences in the secondary structures formed by peptides with different amino acid sequences are due to the great diversity and richness in the properties of amino acids and their side chains (Zhao et al., 2018). Added to this the differences in the non-covalent forces also differ hugely. The length of the hydrophobic chains also affects the diameter and length of the nanostructures resulting from the self-assembly of same amino acid sequences but differing in the number of residues (Han et al., 2011). Reduction in length and diameter of the nanostructures has been observed with an increase in the length of hydrophobic chain from I_3_K to I_5_K. Similarly, A_6_K formed longer nanofibers whereas A_9_K formed shorter nanorods (Cui et al., 2014). The peptides P1, P2, and P3 formed longer tubes whereas P4 (AIIALM), P5 (MVAAVVIA), P6 (MVIIVVIA), and P7 (IIVVII) formed shorter crystals for the same period of incubation (Figure 1A). Cui et al. (2014) also obtained a variety of structures like nanobelts, single and bundled nanofibers, twisted ribbons, helical ribbons, and nanotubes just by switching the amino acid order. Valine has high propensity to form secondary β sheet structure. Cylindrical nanofibers result when sequences changed from VEVE to VVEE as the VV combination induces the hydrophobic collapse as well as the hydrogen bonds existing between the peptides. The tendency of the hydrophobic valine residues at the termini to minimize their contact with water results in the fusion of these peptides resulting in cylindrical curvature as also seen with peptides P1 (MVAAVV) and P2 (MVIIVV) (Figure 1A).

During fibrillogenesis the fibers either become coiled coils or they associate hexagonally to form cylinders of uniform width (Bromley et al., 2010). Crystalline order was observed in fibers in which peptides aligned laterally and longitudinally. The type of nanostructures formed by the peptides is determined by combination of features like hydrogen bonding, hydrophobic interactions and the molecular geometry of amino acid sidechains (Han et al., 2011). The hydrophobicity of the aliphatic peptide chains usually results in the assembly of the peptides into micelles and vesicles (Zhao et al., 2018). The hydrophobic interactions between the amino acids also ensues the lateral association and twisting of peptides resulting in ribbons or tubes or fibers, which are twisted helices. In widely studied peptide amphiphiles (Hartgerink et al., 2002; Hong et al., 2003; Bush and Tanzi, 2008; Krysmann et al., 2008; Han et al., 2011; Zhao et al., 2015, 2018), it is believed that nanostructures are formed by the aggregation of hydrophobic tails that tend to be away from water. The electrostatic interactions between the hydrophilic heads bring about the curvature of those nanostructures. Another vital trigger for self-assembly in a directional manner is the hydrogen bonding that is responsible for β sheet formation along the axial length resulting in tapes, tubes and fibers.

### ThT Assay of Amyloid Formations

Though ThT assay remains one of the widely used techniques to investigate amyloid fibril formations, some studies showed that ThT assay could be inconclusive (Tjernberg et al., 1999; Lindberg et al., 2015). Experimentally treated Aβ (1-42) samples showed abundant fibrils in transmission electron microscopy but their ThT emissions were found to be low. In another study, the nonapeptide Aβ (15-23) (QKLVFFAED) was found to be a very potent ThT binder though it formed only thin flakes as observed in electron microscopy (Tjernberg et al., 1999). The ThT intensities vary significantly even within fibrils of isoforms of the same amyloid protein depending on the fibril structure. It has also been found that though ThT analogs bound to amyloid oligomers they recorded low fluorescence emissions and it was proposed that in spite of having higher binding affinities for ThT the amyloid species might be having lesser number of binding sites (Lindberg et al., 2015).

In the cluster of peptides in this study, though there is an increase in ThT fluorescence initially, after reaching a peak of fluorescence intensity there is a decline in the ThT curves of almost all the peptides (Figure 2). This might be due to fibril breakage which is an important secondary process that governs the fibril formation (Hellstrand et al., 2010). A similar pattern has been observed in the fibrillar oligomers of Aβ which are rich in β sheet content but bind weakly to ThT. It was suggested that these fibrillar oligomers may grow only to a certain height and then split, affecting the binding of ThT to them (Wu et al., 2010).

Thus, despite its widespread use, the binding mode of ThT to amyloid fibrils and the origin of its enhanced fluorescence upon binding remain debated (Necula et al., 2007; Lindberg et al., 2015). It was concluded that ThT assay needs to be complemented with techniques like electron microscopy while monitoring amyloid fibril formations. Nevertheless, enhancement of ThT fluorescence is a general feature of amyloid aggregation (Hellstrand et al., 2010; Sharma et al., 2015), and it is used routinely for detecting amyloid aggregation.

### β Sheet Conformations of the Peptides

Much of the amyloid fibrils stability is attributed to the helical β sheets that are continuously hydrogen bonded over long distances along the fibril axis and this stability of amyloid fibrils poses a major challenge in amyloid diseases (Zandomeneghi et al., 2004). It has been shown that low nanomolar concentrations of Aβ are sufficient to enter the cells, if they are rich in β sheet content (Jin et al., 2016). On the contrary, uptake of monomers depended on a minimal threshold of concentration or higher nanomolar concentrations. FT-IR analysis of our cluster of peptides showed that they are all β sheet formers (Figure 3). The β sheet conformations are vital in ensuing the amyloid aggregation and fibril formation (Luhrs et al., 2005). The abnormal aggregates formed by a variety of amyloid proteins involved in different amyloid diseases all have β sheet structure in common (Glabe, 2008). Our peptides can thus be prospective models to explore the amyloid structural relations to cellular uptake and cytotoxicity, and also therapeutic interventions for amyloid diseases.

### Inhibitors of Amyloid Formations

Quinones are oxidative intermediates of polyphenols and through molecular dynamics (MD) simulations it was demonstrated that they break the intermolecular hydrogen bonds of cross β- strands in oligomers and fibrils. This is possible due to their more favorable polar interactions with the peptide backbone by engaing their carbonyl hydrogens, oxygens, and amides in tight hydrogen bonds and through π^+^δ^−^ interactions between the quinolic ring and the peptide backbone (Convertino et al., 2009). Many compounds like quinones, epigallocatechine gallate, curcumin, and resveratrol have been evaluated for their ability to affect the amyloid aggregation along with small peptides (Dolphin et al., 2006) or oligomers or antibodies specific for different amyloid aggregates (Scheidt et al., 2019). Polyphenols from wine also were found to be effective inhibitory agents of amyloid formation, fibril extension and destabilization. Mancini et al. (2018) investigated the ability of different components of instant coffee to inhibit the fibrillization of Aβ and tau proteins and found that among them phenylindane has the most potent aggregation inhibition activity. Another investigation on the ability of polyphenolic amyloid inhibitors has shown that 1,4-benzoquinone and 1,4-napthoquinone are potentially active against oligomerization of insulin into amyloid fibrils (Gong et al., 2014). The TEM (transmission electron micrscopy) images of insulin treated with napthoquinone showed amorphous aggregates and not the fibrils found in the control protein sample.

In conclusion, investigations on our peptide cluster show that they are all potential Aβ sheet mimics that can be used to understand the mechanisms involved in the formation of different amyloid polymorphs from peptides of different amino acid sequences. They can be very useful models in testing prospective therapeutic strategies for various amyloid diseases. Usually therapeutic research for most of the illnesses is directed at a single compound or infection. Protein diseases are complex processes ensued by conformational change in a protein as is the case with AD. More and more studies on tracking the progress of amyloid into different forms generated a multitude of knowledge but the progression of Aβ into cytotoxic entities remains elusive (Cerasoli et al., 2015). Recently Galzitskaya et al. (2018) have proposed that the treatement of amyloidosis needs to be personalized based on the changed proteins/peptides at genetic level as Alzheimer's disesase is known to be accelerated by various mutations in Aβ peptides that might result in different morphologies of amyloid fibrils in different patients. This is evident in our observation that different peptide sequnces gave different morphologies as seen in their SEM images. They also showed different binding capacities to ThT. The inhibitory action of HNQ has also been different on different peptides some of which showed amorphous aggregates and some others lesser density of shortened amyloid aggregates on treating with HNQ. Understanding the amyloid fibril arrangements during maturation processes and the resulting fibril polymorphisms are crucial for designing therapeutics for amyloid diseases (Psonka-Antonczyk et al., 2016) and these peptide clusters can be effective tools in such studies.

## Data Availability Statement

The raw data supporting the conclusions of this article will be made available by the authors, without undue reservation. Sample scanning electron micrographs of peptides P1 to P7 at different resolutions are included in Supplementary Information.

## Author Contributions

LS conceived, helped design experiments, supervised the research, and helped write and edit the manuscript. RM designed and performed the experiments, analyzed the data, and wrote and edited the manuscript. MY conducted SEM experiments and helped analyze the results. All authors contributed to the article and approved the submitted version.

## Conflict of Interest

The authors declare that the research was conducted in the absence of any commercial or financial relationships that could be construed as a potential conflict of interest.

## Supplementary Material

Figure S1 Scanning electron micrographs of peptides P1 to P6 at different resolutions.

Figure S2 Scanning electron micrographs of peptide P7 at different resolutions.
