# Supplementary figures and images for: Nanostructures Formed by Custom-Made Peptides Based on Amyloid Peptide Sequences and Their Inhibition by 2-Hydroxynaphthoquinone
Source: Front Chem. 2020 Aug 6;8:684. doi: 10.3389/fchem.2020.00684 (PMC7424059; doi:10.3389/fchem.2020.00684)

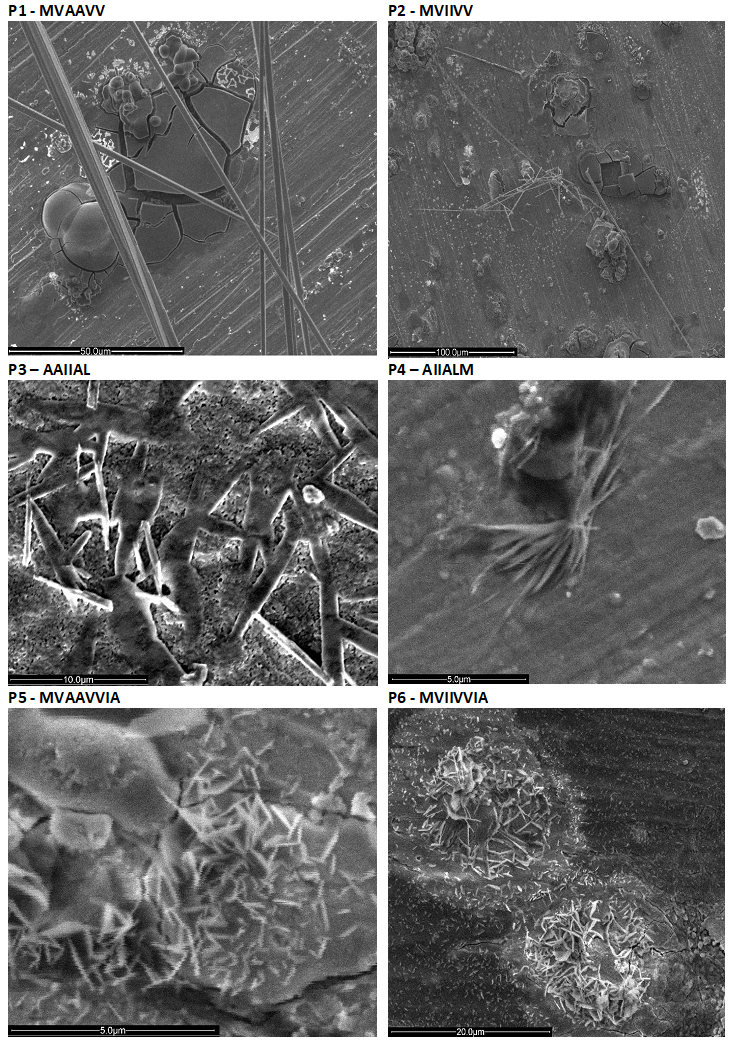

Supplement: Figure S1 — Scanning electron micrographs of peptides P1 to P6 at different resolutions. [file Image_1.tif]

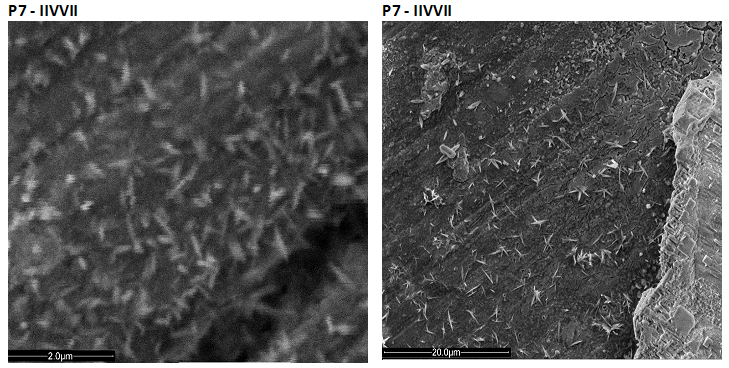

Supplement: Figure S2 — Scanning electron micrographs of peptide P7 at different resolutions. [file Image_2.tif]
